# Supplementary material for: Cytotoxic, anti-inflammatory, antioxidant, and anti-glyoxalase-I evaluation of chelating substances: In silico and in vitro study
Source: PLoS One. 2025 Oct 15;20(10):e0333405. doi: 10.1371/journal.pone.0333405 (PMC12527173; doi:10.1371/journal.pone.0333405)
Supplement: S1 File — (DOCX) [file pone.0333405.s001.docx]

**Supporting Information (S1 File):**

**#1 4-hdroxy estrdiol**

**Table 1** Data of enzyme inhibition assay of **(4-hdroxy estrdiol)**

| **Concentrations μM** | **Trial one (%inhibtion)** | **Trial two (%inhibtion)** | **Trial three (%inhibtion)** |
| --- | --- | --- | --- |
| 50 | 101.9366 | 100.8803 | 101.4085 |
| 25 | 101.9366 | 101.9366 | 103.5211 |
| 12.5 | 99.82394 | 98.69718 | 99.29577 |
| 6.25 | 95.59859 | 97.71127 | 96.12676 |
| 3.125 | 92.95775 | 96.12676 | 95.59859 |
| 1.562 | 99.82394 | 91.37324 | 94.01408 |
| 0.7812 | 80.80986 | 82.92254 | 81.33803 |
| 0.3906 | 69.71831 | 67.07746 | 66.02113 |
| 0.1953 | 57.57042 | 55.45775 | 59.15493 |
| 0.09765 | 17.95775 | 21.65493 | 20.59859 |

**Table 2** Descriptive statistics of **(4-hdroxy estrdiol)**

| **Number of values** | 10 |
| --- | --- |
|  |  |
| **Minimum** | 20.07 |
| **Maximum** | 102.5 |
| **Range** | 82.39 |
|  |  |
| **Mean** | 81.63 |
| **Std. Deviation** | 26.43 |
| **Std. Error of Mean** | 8.358 |

**#2 Shikonine**

**Table 3** Data of enzyme inhibition assay of **(shikonine)**

| **Concentrations μM** | **Trial one (%inhibtion)** | **Trial two (%inhibtion)** | **Trial three (%inhibtion)** |
| --- | --- | --- | --- |
| 50 | 104.1714 | 96.92462 | 100.9747 |
| 25 | 72.47169 | 74.16078 | 73.87914 |
| 12.5 | 59.39086 | 65.10601 | 61.01365 |
| 6.25 | 34.7911 | 45.79329 | 51.26706 |
| 3.125 | 16.91982 | 23.78681 | 30.01949 |

**Table 4** Descriptive statistics of (**shikonine)**

| **Number of values** | 5 |
| --- | --- |
|  |  |
| **Minimum** | 23.58 |
| **Maximum** | 100.7 |
| **Range** | 77.11 |
|  |  |
| **Mean** | 60.71 |
| **Std. Deviation** | 29.25 |
| **Std. Error of Mean** | 13.08 |

**#3 Tolcopone**

**Table 5** Data of enzyme inhibition assay of **(tolcopone)**

| **Concentrations μM** | **Trial one (%inhibtion)** | **Trial two (%inhibtion)** | **Trial three (%inhibtion)** |
| --- | --- | --- | --- |
| 50 | 96.09528 | 97.57067 | 102.2999 |
| 25 | 78.13354 | 82.11131 | 80.91076 |
| 12.5 | 54.17181 | 60.90989 | 56.53174 |
| 6.25 | 27.95783 | 39.70848 | 36.29255 |
| 3.125 | 12.33893 | 19.39046 | 20.42318 |

**Table 6** Descriptive statistics of **(tolcopone)**

| **Number of values** | 5 |
| --- | --- |
|  |  |
| **Minimum** | 17.38 |
| **Maximum** | 98.66 |
| **Range** | 81.27 |
|  |  |
| **Mean** | 57.66 |
| **Std. Deviation** | 32.97 |
| **Std. Error of Mean** | 14.75 |

**#4** **Quinaziarin**

**Table 7** Data of enzyme inhibition assay of **(Quinaziarin)**

| **Concentrations μM** | **Trial one (%inhibtion)** | **Trial two (%inhibtion)** | **Trial three (%inhibtion)** | |
| --- | --- | --- | --- | --- |
| 50 | 55.50636 | 59.85751 | 55.34625 |  |
| 25 | 42.74809 | 46.56234 | 43.55518 |  |
| 12.5 | 37.15267 | 35.3715 | 41.76917 |  |
| 6.25 | 32.56997 | 28.56997 | 33.65628 |  |
| 3.125 | 22.83206 | 19.54962 | 28.60152 |  |

**Table 8** Descriptive statistics of (**Quinaziarin)**

| **Number of values** | 5 |
| --- | --- |
|  |  |
| **Minimum** | 23.66 |
| **Maximum** | 56.90 |
| **Range** | 33.24 |
|  |  |
| **Mean** | 38.91 |
| **Std. Deviation** | 12.64 |
| **Std. Error of Mean** | 5.654 |

**#5 Hispidine**

**Table 9** Data of enzyme inhibition assay of **(hispidine)**

| **Concentrations μM** | **Trial one (%inhibtion)** | **Trial two (%inhibtion)** | **Trial three (%inhibtion)** |
| --- | --- | --- | --- |
| 50 | 53.482 | 44.27958 | 54.52541 |
| 25 | 47.93531 | 32.44826 | 43.53861 |
| 12.5 | 29.52529 | 19.1722 | 34.32463 |
| 6.25 | 19.07186 | 9.996094 | 23.96799 |
| 3.125 | 10.90332 | 4.724717 | 20.22999 |

**Table 10** Descriptive statistics of **(hispidine)**

| **Number of values** | 5 |
| --- | --- |
|  |  |
| **Minimum** | 11.95 |
| **Maximum** | 50.76 |
| **Range** | 38.81 |
|  |  |
| **Mean** | 29.88 |
| **Std. Deviation** | 16.14 |
| **Std. Error of Mean** | 7.217 |

**#6 Trichostatn**

**Table 11** Data of enzyme inhibition assay of **(trichostatn)**

| Concentrations μM | Trial one (%inhibtion) | Trial two (%inhibtion) | Trial three (%inhibtion) |
| --- | --- | --- | --- |
| 50 | 52.07597 | 51.07399 | 38.83721 |
| 25 | 39.69181 | 38.35004 | 33.72093 |
| 12.5 | 29.40813 | 24.97772 | 24.65116 |
| 6.25 | 22.26148 | 16.46221 | 14.18605 |
| 3.125 | 13.20671 | 2.774861 | 7.44186 |

**Table 12** Descriptive statistics of **(trichostatn)**

| **Number of values** | 5 |
| --- | --- |
|  |  |
| **Minimum** | 7.808 |
| **Maximum** | 47.33 |
| **Range** | 39.52 |
|  |  |
| **Mean** | 27.27 |
| **Std. Deviation** | 15.61 |
| **Std. Error of Mean** | 6.980 |

**#7 Calceolarioside A**

**Table 13** Data of enzyme inhibition assay of **(Calceolarioside A)**

| Concentrations μM | Trial one (%inhibtion) | Trial two (%inhibtion) | Trial three (%inhibtion) |
| --- | --- | --- | --- |
| 50 | 55.55556 | 53.46319 | 55.98147 |
| 25 | 35.28265 | 27.48836 | 29.55013 |
| 12.5 | 25.34113 | 11.96189 | 20.91323 |
| 6.25 | 14.42495 | 9.060927 | 9.014322 |
| 3.125 | 15.39961 | 7.904023 | 6.697557 |

**Table 14** Descriptive statistics of **(Calceolarioside A)**

| **Number of values** | 5 |
| --- | --- |
|  |  |
| **Minimum** | 10.00 |
| **Maximum** | 55.00 |
| **Range** | 45.00 |
|  |  |
| **Mean** | 25.20 |
| **Std. Deviation** | 18.64 |
| **Std. Error of Mean** | 8.337 |

**#8 Myrctine (Positive control)**

**Table 15** Data of enzyme inhibition assay of **(myrctine)**

| Concentrations μM | Trial one (%inhibtion) | Trial two (%inhibtion) | Trial three (%inhibtion) |
| --- | --- | --- | --- |
| 50 | 110.27 | 108.9326 | 101.5632 |
| 25 | 101.56 | 104.9129 | 104.243 |
| 12.5 | 94.19 | 95.53372 | 94.19384 |
| 6.25 | 66.06 | 67.39616 | 69.40598 |
| 3.125 | 43.95 | 47.96784 | 44.61813 |
| 50 | 14.47 | 16.48057 | 17.15051 |

**Table 16** Descriptive statistics of **(myrctine)**

| **Number of values** | 6 |
| --- | --- |
|  |  |
| **Minimum** | 16.03 |
| **Maximum** | 106.9 |
| **Range** | 90.89 |
|  |  |
| **Mean** | 72.38 |
| **Std. Deviation** | 36.28 |
| **Std. Error of Mean** | 14.81 |
