# Supplementary material for: Cytotoxic, anti-inflammatory, antioxidant, and anti-glyoxalase-I evaluation of chelating substances: In silico and in vitro study
Source: PLoS One. 2025 Oct 15;20(10):e0333405. doi: 10.1371/journal.pone.0333405 (PMC12527173; doi:10.1371/journal.pone.0333405)
Supplement: Attachment — Submitted filename: PLOS_REVIEWER COMMENT_PONE-D_25_10069.pdf [file pone.0333405.s002.pdf]

Reviewer Comments:

Manuscript Number PONE-D-25-10069

Title: Cytotoxic, Anti-inflammatory, Antioxidant, and Anti-Glyoxalase-I Evaluation of Chelating Substances; in silico and in vitro Study

The manuscript presents a comprehensive study aimed at evaluating the cytotoxic, anti-inflammatory, antioxidant, and anti-glyoxalase-I properties of chelating substances through both in silico and in vitro methodologies. This dual approach is commendable and provides a robust framework for understanding the multifaceted roles these compounds may play in therapeutic applications. The objectives are clear and align well with the significant scientific interest in chelating agents and their potential health benefits.

Strengths:

1. Innovative Approach: The integration of in silico and in vitro studies offers a powerful platform for validating findings and enhances the reliability of the results. The computational modeling adds depth to the understanding of molecular interactions and potential mechanisms of action.

2. Well-Defined Methodology: The experimental design is clearly articulated, with appropriate controls and replicates. The selection of assays for assessing cytotoxicity, inflammation, and oxidative stress is appropriate and well-justified.

3. Statistical Analysis: The use of statistical methods to analyze and interpret the data is commendable. This adds rigor to the findings and provides a baseline for future studies.

4. Relevance of Findings: The study addresses critical health-related issues, including inflammation and oxidative stress. The exploration of anti-glyoxalase-I activity is particularly relevant, given the implications for various metabolic disorders.

Areas for Improvement:

1. Literature Review: While the manuscript cites relevant studies, a more thorough discussion of the existing literature would strengthen the background. Highlighting how this study builds upon or contrasts with previous work could provide greater context for your findings.

2. In Silico Methods: The methodologies employed in the in silico portion of the study should be described in greater detail. Specific algorithms used for molecular docking and the criteria for evaluating binding affinities should be included to allow replication and validation by other researchers

3. Data Presentation: Figures and tables are pivotal for clarity. Enhancing the quality of the figures, particularly those depicting dose-response curves and molecular structures, would improve the readability of the results. Consider including error bars in your graphical data to represent variability.

4. Discussion of Mechanisms: The discussion would benefit from a deeper exploration of the mechanisms behind the observed anti-inflammatory and antioxidant effects. Speculative mechanisms could be suggested based on existing literature, helping to position the findings within the broader context of biochemical pathways

5. Conclusions and Future Directions: The conclusion section could be expanded to outline potential future research avenues that could stem from this work. Discussing the implications of the findings for clinical applications would be beneficial as well.

Minor Comments:

- Check for typographical errors and ensure consistency in terminology throughout the manuscript.

- Ensure that all references are formatted according to the journal's guidelines.

- Clarify any abbreviations at first mention in the text to maintain reader accessibility.

Overall Evaluation:

This manuscript is a valuable contribution to the field, with important implications for the design of therapeutic agents based on chelating substances. Addressing the comments outlined above will strengthen the manuscript significantly and enhance its impact in the scientific community. I recommend a minor revision before considering publication.

If possible for authors kindly cite below papers in the manuscript:

1. Cheng, Y., Wang, L., Zhang, S., Jian, W., Zeng, B., Liang, L.,... Deng, Z. (2024). The Investigation of Nfκb Inhibitors to Block Cell Proliferation in OSCC Cells Lines. Current Medicinal Chemistry. doi: 10.2174/0109298673309489240816063313

2. Li, R., Luo, P., Guo, Y., He, Y., & Wang, C. (2024). Clinical features, treatment, and prognosis of SGLT2 inhibitors induced acute pancreatitis. Expert Opinion on Drug Safety, 1-5. doi: https://doi.org/10.1080/14740338.2024.2396387

3. Kang, L., Gao, X., Liu, H., Men, X., Wu, H., Cui, P.,... Yan, J. (2018). Structure–activity relationship investigation of coumarin–chalcone hybrids with diverse side-chains as acetylcholinesterase and butyrylcholinesterase inhibitors. Molecular Diversity, 22(4), 893-906. doi: 10.1007/s11030-018-9839-y

4. Shi, S., Li, K., Peng, J., Li, J., Luo, L., Liu, M.,... Cai, W. (2022). Chemical characterization of extracts of leaves of Kadsua coccinea (Lem.) A.C. Sm. by UHPLC-Q-Exactive Orbitrap Mass spectrometry and assessment of their antioxidant and anti-inflammatory activities. Biomedicine & Pharmacotherapy, 149, 112828. doi: https://doi.org/10.1016/j.biopha.2022.112828

5. Zhou, J., Guo, Z., Peng, X., Wu, B., Meng, Q., Lu, X.,... Guo, T. (2025). Chrysotoxine regulates ferroptosis and the PI3K/AKT/mTOR pathway to prevent cervical cancer. Journal of Ethnopharmacology, 338, 119126. doi: https://doi.org/10.1016/j.jep.2024.119126
